# Retraction: Grape Seed Proanthocyanidins Inhibit Melanoma Cell Invasiveness by Reduction of PGE_2_ Synthesis and Reversal of Epithelial-to-Mesenchymal Transition

**DOI:** 10.1371/journal.pone.0210343

**Published:** 2018-12-31

**Authors:** The PLOS ONE Editors

Following publication of this article [1], concerns were raised about Figure 5B. For each experimental condition, Vimentin and Fibronectin immunofluorescence panels in the figure appear to be derived from the same original image. The University of Alabama at Birmingham confirmed that original data and records needed to clarify the experiment presented in these panels are not available.

Following a joint investigation by the Birmingham VA Medical Center and the University of Alabama at Birmingham, the institutions requested retraction of this article, as the conclusions could not be supported by available data. In line with the institutions’ investigation and recommendation, the *PLOS ONE* Editors retract this article based upon the unavailability of original data and records and the ambiguous identification of samples and treatments.

The authors did not comment on the retraction decision.
